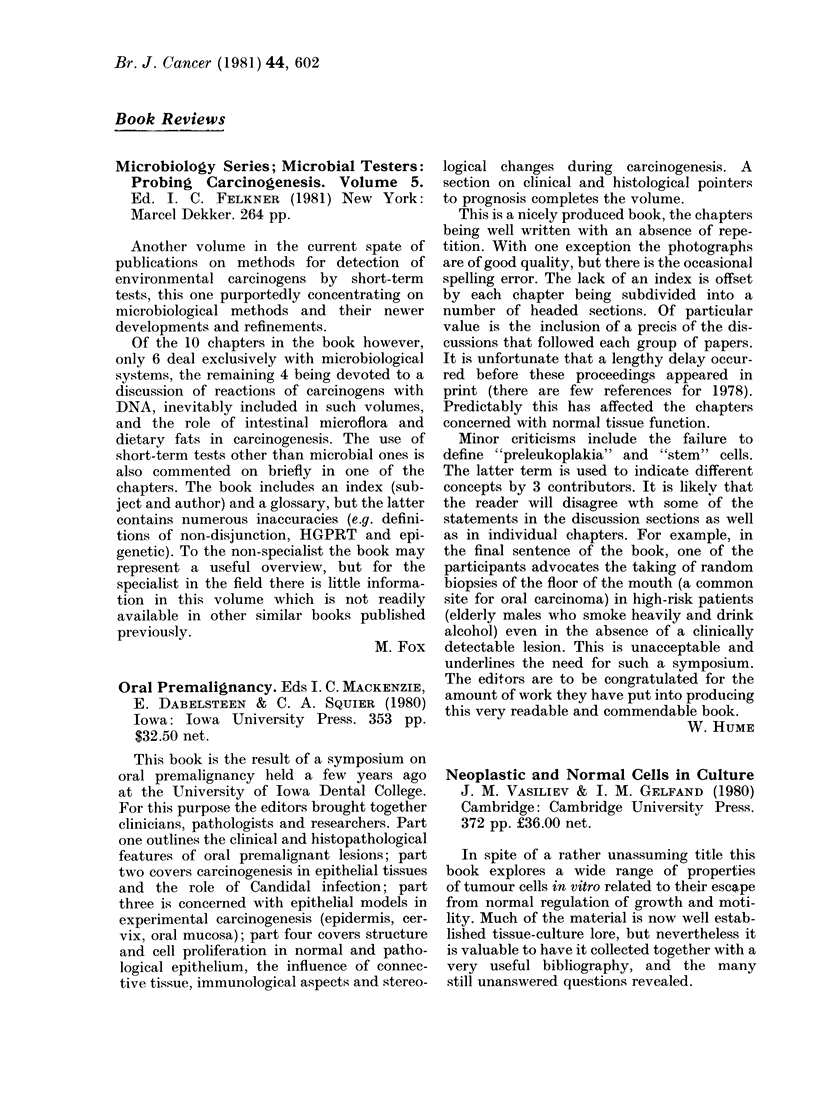# Oral Premalignancy

**Published:** 1981-10

**Authors:** W. Hume


**Oral Premalignancy.** Eds I. C. Mackenzie, E. Dabelsteen & C. A. Squier (1980) Iowa: Iowa University Press. 353 pp. $32.50 net.

This book is the result of a symposium on oral premalignancy held a few years ago at the University of Iowa Dental College. For this purpose the editors brought together clinicians, pathologists and researchers. Part one outlines the clinical and histopathological features of oral premalignant lesions; part two covers carcinogenesis in epithelial tissues and the role of Candidal infection; part three is concerned with epithelial models in experimental carcinogenesis (epidermis, cervix, oral mucosa); part four covers structure and cell proliferation in normal and pathological epithelium, the influence of connective tissue, immunological aspects and stereological changes during carcinogenesis. A section on clinical and histological pointers to prognosis completes the volume.

This is a nicely produced book, the chapters being well written with an absence of repetition. With one exception the photographs are of good quality, but there is the occasional spelling error. The lack of an index is offset by each chapter being subdivided into a number of headed sections. Of particular value is the inclusion of a precis of the discussions that followed each group of papers. It is unfortunate that a lengthy delay occurred before these proceedings appeared in print (there are few references for 1978). Predictably this has affected the chapters concerned with normal tissue function.

Minor criticisms include the failure to define "preleukoplakia" and "stem" cells. The latter term is used to indicate different concepts by 3 contributors. It is likely that the reader will disagree wth some of the statements in the discussion sections as well as in individual chapters. For example, in the final sentence of the book, one of the participants advocates the taking of random biopsies of the floor of the mouth (a common site for oral carcinoma) in high-risk patients (elderly males who smoke heavily and drink alcohol) even in the absence of a clinically detectable lesion. This is unacceptable and underlines the need for such a symposium. The editors are to be congratulated for the amount of work they have put into producing this very readable and commendable book.

W. Hume